# Effectiveness of levofloxacin in the induction of chemotherapy in high-risk acute lymphoblastic leukaemia in children in a developing country

**DOI:** 10.3332/ecancer.2023.1606

**Published:** 2023-09-25

**Authors:** Areeba Ejaz, Asim Fakhruddin Belgaumi, Syed Ejaz Alam, Mohammad Shamvil Ashraf, Mohammad Rafie Raza

**Affiliations:** 1 Indus Hospital and Health Network, Plot C-76, Sector 31/5, Opposite Darussalam Society, Korangi Crossing, Karachi, Pakistan; 2 Aga Khan University, Stadium Road, ​PO Box 350​0, Karachi 7480​0, Pakistan; 3 Pakistan Medical and Research Council, PMRC Center for Hepatology and Gastroenterology, JPMC, Rafiqi H J Rd, Cantonment Karachi, 75510, Karachi City, Sindh, Pakistan

**Keywords:** leukaemia, children, prophylaxis, levofloxacin, infections

## Abstract

**Background:**

Infections significantly predominate during induction chemotherapy for acute lymphoblastic leukaemia (ALL) in children. Antibacterial prophylaxis is one strategy that lowers the risk of these infections. This study evaluates the role of levofloxacin prophylaxis on the frequency of infections, febrile neutropenia (FN) and outcomes associated with it along with the development of drug-resistance.

**Subject and methods:**

This was a single-centre cohort study in which the data were collected from electronic health records between two cohorts of high-risk ALL patients in the induction phase: the first one before the initiation of levofloxacin prophylaxis and the second was after the implementation of levofloxacin prophylaxis. The variables were compared between both the groups and odds ratios were calculated for clinical outcomes.

**Results:**

Out of 227 patients, 115 were given levofloxacin prophylaxis and 112 were in the no prophylaxis group. Both cohorts were similar in demographic factors, treatment regimen and supportive care services. There was a significant difference in total in-patient admissions along with FN admissions (*p* = 0.026). Microbiologically documented infections and infection-related critical interventions were significantly higher in the no prophylaxis group (*p* < 0.05). Odds ratios with a 95% confidence interval were applied to both groups for clinical outcomes in patients with and without FN which also illustrated similar results. Overall mortality and drug resistance patterns were similar among both groups.

**Conclusion:**

This study emphasised that levofloxacin is effective in reducing inpatient admissions with FN and its complications but did not affect the drug-resistance pattern. Long-term monitoring for antibiotic resistance is mandatory.

## Introduction

Treatment-related mortality and morbidity are one of the most important aspects of acute lymphoblastic leukaemia (ALL) management, in which infections play a major role; the reported mortality rate in paediatric ALL patients in developed countries, due to infections, is around 4% [1–4]. However, it remains relatively high in low- and middle-income countries (LMICs). Marwaha* et al* [5] from India, as well as Jabeen *et al* [6] from Pakistan, reported between 10% and 20% mortality during induction. Similarly, results from another local study in Pakistan showed a 14% induction mortality rate with upfront multi-agent chemotherapy which was lowered to 9% when prednisolone prophase was administered for 1 week instead of upfront multi-agent chemotherapy [7]. This higher treatment-related mortality, particularly during induction, is a key concern for underdeveloped countries. The reasons are many, including a late presentation with a higher illness burden, a high prevalence of infection, malnourishment and poor supportive care measures.

Febrile neutropenia (FN) is one of the known consequences of chemotherapy in haematologic malignancies [8]. Patients with FN often experience bacterial infections that, although not yet confirmed through a blood culture, have the potential to progress to septicaemia, resulting in increased treatment-related morbidity and mortality. Without preventive measures, 48%–60% of FN patients acquire infections, and 16%–20% of profoundly neutropenic (neutrophil counts < 0.1 × 10^9^ cells/L) patients experience bacteraemia [9]. Trimethoprim-sulfamethoxazole has been used as antimicrobial prophylaxis in routine clinical practice though fluoroquinolones are another appealing option as they have a broader antimicrobial spectrum [10]. When used as antibiotic prophylaxis, fluoroquinolones may help decrease infections in patients with FN, but inculcating this as standard practice is still controversial as some studies have shown benefits yet others do not show similar results [11, 12].

In adult cancer patients undergoing rigorous chemotherapy, antibiotic prophylaxis has demonstrated decreased bacteraemia and enhanced survival [13]. A study conducted in Saudi Arabia on children with ALL reported a decreased frequency of bloodstream infections (BSIs) with ciprofloxacin prophylaxis during delayed intensification [14]. In a large study conducted at St. Jude Children’s Research Hospital, reduced rates of FN, microbiologically documented infection (MDI), likely bacterial infection (LBI), and BSIs were observed after levofloxacin prophylaxis during induction therapy for ALL [15]. Another multicentre, randomised, open-label phase 3 trial (ACCL0934) conducted by the Children’s Oncology Group concluded that the receipt of levofloxacin prophylaxis compared to no prophylaxis in children with acute leukaemia who received intensive chemotherapy led to significant reductions in bacteraemia [16]. Recently published clinical practice guidelines suggest a weak recommendation for antibacterial prophylaxis in children with acute myeloid leukaemia (AML) and relapsed ALL, with levofloxacin being the preferable antibacterial drug also in addition weak recommendations were given against the usual use of systemic antibiotic prophylaxis for children having induction chemotherapy for ALL due to scarce data on children [17].

There is limited published data regarding antibacterial prophylaxis in the paediatric populations with haematologic malignancies. Frequent inpatient admissions due to FN and deaths due to infections impose a major hurdle in the treatment of these patients in LMIC. Despite similar treatment protocols as that of western countries, chemotherapy-induced toxic effects are more severe in resource-limited settings. Because of persistent neutropenia, these harmful effects include serious infections and infection-related mortality. This study explores the role of antibacterial prophylaxis on the frequency of infections, FN and complications associated with it, like septicaemia and septic shock and treatment-related mortality, and it also addresses the issue of drug resistance to fluoroquinolones once the prophylaxis has been started. Finally, this study might help to establish standard guidelines for antibiotic prophylaxis in our setting.

## Materials and methods

This study was conducted at The Indus Hospital (TIH), by the Paediatric Oncology Department, Karachi, Pakistan. TIH is a charitable hospital in which most patients with poor socioeconomic status are served, as the quality healthcare provided is free of cost. The Paediatric Haematology Oncology Department of TIH is one of the largest in the country, seeing >800 new patients per year. Induction therapy is provided on an outpatient basis as inpatient capacity is limited and most of the time is occupied with admissions secondary to infections because of FN and drug toxicities.

A modified approach of prednisolone prophase without any other chemotherapeutic agent is adapted to offer minimally toxic treatment for a week to both groups except for those patients who present with mediastinal mass or leukostasis [18]. The objective is to gradually decrease the leukaemia burden, thereby reducing the risk of toxic death due to tumour lysis or prolonged and severe neutropenia, especially in the settings of LMICs. This also allows nutritional rehabilitation and treatment of coexistent infections. On day 8 of treatment prophase response assessment was performed. The criteria of absolute blast count of <1,000 versus ≥1,000/μL on peripheral blood was used as good versus poor response, respectively. Poor responders were assigned to high-risk group.

According to the risk group, according to the National Cancer Institute risk, standard and high (HR), a multi-agent chemotherapeutic protocol was started, which included three or four cytotoxic drugs, respectively, with the difference of anthracycline treatment given to the HR group in induction phase using the modified Berlin-Frankfurt-Münster ALL protocol. Patients with CNS-3, age > 10 years, and those with unfavourable cytogenetics (Hypodiploidy, *MLL* rearrangements, *BCR-ABL* translocation) were considered HR.

As per our institutional policy antibiotic prophylaxis was not a part of high-risk leukaemia protocol before November 2018, so data from one cohort, i.e., no prophylaxis group will be collected from electronic medical records from November 2017 to 2018. In 2019, our institute added levofloxacin prophylaxis during the induction phase as part of the leukaemia protocol, based on international studies that were conducted in that time period. These studies showed favourable outcomes with the addition of levofloxacin prophylaxis [14–16]. Primary prophylaxis was assessed from the commencement of multi-agent induction chemotherapy until the onset of FN or infection, or until the end of induction therapy (first clinical event). Dosing for levofloxacin prophylaxis was as follows: age <5 years, 10 mg/kg PO every 12 hours (max. 250 mg every 12 hours); age ≥5 years, 10 mg/kg PO daily (max. 500 mg daily). The data of the levofloxacin prophylaxis cohort were collected after institutional review board approval, from December 2020 to 2021. A sample size of 226, with 113 in each arm, was targeted.

Patients of both genders who are diagnosed as high-risk ALL from the age of 1 year and above during induction therapy and also those who were admitted to the hospital with FN or any suspected infection were included in the study. Those who are under the age of 1 year and who were admitted for any reason other than FN or any suspected infection were excluded. Patients with ALL who already had infection or culture-positivity before initiation of chemotherapy were also not included. Those patients who developed FN or suspected infection during the first 7 days of induction were also excluded from both groups due to inadequate time for the effectiveness of primary prophylaxis.

The Common Terminology Criteria for Adverse Events, version 5.0 [19], were used to capture all infection-related adverse events in the study, and standard definitions were modulated to further categorise episodes as per our institutional standards and limitations as depicted in Table 1.

Routine radiological investigations and blood samples that were drawn from all patients during the inpatient stay were considered. Inpatient admissions due to FN or any infective cause were considered our primary outcome. The type of infection was labelled as per the operational definitions given above and was sorted into groups of clinically documented infection (CDI), LBI, MDI and BSI, as defined above. The clinical implications of these infections like sepsis, severe sepsis, sentinel events or any urgent intervention required were measured for statistical analysis. These variables were compared between two cohorts of ALL patients on induction chemotherapy, one with levofloxacin prophylaxis and the other without levofloxacin prophylaxis.

Clinical management of infection was at the discretion of the oncology team attending physician. The standard empirical antibiotic administered for fever and neutropenia was piperacillin/tazobactam and amikacin. If there was a concern for an intra-abdominal focus, the substitution of meropenem for piperacillin/tazobactam was done. Vancomycin was added when there was a concern for skin infection, central line infection or if the patient has a history of colonisation or infection with methicillin-resistant *Staphylococcus aureus*. Among microbiologically documented FN episodes in our oncology centre in a year, the most identified pathogens were bacteria 78.6%, followed by viral infections 15%, fungus 6% and *Mycobacterium tuberculosis* (one patient 0.4%). Gram-negative bacteria were the most common pathogen (49%), followed by Gram-positive bacteria (23.4%). The most common bacteria which were isolated were *Escherichia coli* 24.7%, *Klebsiella pneumoniae* 12.3%, and *Acinetobacter* 5% and *Pseudomonas* 4.5%. Drug resistance to quinolones is 51.5% amongst these pathogens.

The decision to discharge was made on the improvement in the clinical condition of the patient, completion of total antibiotic days as per the infectious disease department and the rising trend of neutrophil counts. The *Pneumocystis jiroveci* pneumonia prophylaxis was trimethoprim-sulfamethoxazole as per our institutional standards. Fluoroquinolone sensitivity or resistance was measured from the culture and sensitivity report. Drug resistance patterns were also compared.

### Data analysis

Data was entered and analysed using Statistical Package for Social Sciences (SPSS) version 19.0. Mean ± SD was computed for all the quantitative variables such as age, weight and height, etc. Frequency and percentage were computed for all the categorical variables like gender, category of neutropenia, etc. An independent sample *T*-test was applied for all quantitative variables. Chi-square test/Fisher’s exact test was applied to qualitative variables. Odds ratios with a 95% confidence interval (CI) were used to assess the association between the prophylaxis group and clinical outcomes. *p*-value < 0.05 was considered statistically significant.

## Results

The total number of patients who fulfilled the inclusion criteria was 234; out of which 4 patients abandoned treatment in total, 1 patient left against medical advice in the no prophylaxis group due to non-compliance while two patients were also excluded as they were lost to follow-up. Hence, 115 patients were in the levofloxacin prophylaxis group and 112 were in the no prophylaxis group. The data of 115 patients were prospectively collected, while the data were extracted retrospectively for the comparison group of 112 patients. Those who were admitted without any suspected infection or had an infection before the opportunity to start prophylaxis, or who had an infection before starting chemotherapy were not considered eligible. Age groups, gender and body mass index were similar between both groups as shown in Table 2.

Table 2 also highlighted that the total number of hospital admissions was significantly reduced in the prophylaxis group (*p* = 0.026). The average duration of hospital admission for patients was shorter in patients who received prophylaxis as compared to those who did not (2 versus 7 days; *p* = 0.016). Patients who remained admitted for >7 days were mostly for the completion of the total antibiotic duration. However, no significant difference in readmission in both the groups. This study also found a significant decline in the total number of admissions with FN in the levofloxacin group (*p* = 0.008).

Table 3 showed that LBIs were higher in the no prophylaxis group, but statistical significance was not found. There is a statistically significant difference in MDIs; 32.9% in patients without prophylaxis and 16.4% in those who were receiving levofloxacin prophylaxis (*p* = 0.035). In the prophylaxis group, only one patient had a BSI. The need for urgent intervention like fluid resuscitation and high-density unit (HDU)/ICU admissions was significantly higher in the no prophylaxis group as shown in Table 3 (*p* = 0.002). The number of patients in the levofloxacin group (*n* = 5) who underwent sepsis and septic shock was lesser than in the no prophylaxis group (*n* = 10) but statistically, this was not significant. There was no statistical difference in mortality due to infection-related complications in both the groups.

Table 3 also depicted the details of cultures and drug-resistance patterns observed in admitted patients. A total of 25 patients had a culture-positive infection of which 6 out of 55 patients had positive cultures in the levofloxacin group and 19 out of 70 patients in the no prophylaxis group (*p* = 0.030). The incidence of gram-negative organisms was equally high in both groups. Fluoroquinolone resistance remained a bit high, and more patients had multidrug-resistant pathogens in the levofloxacin group although statistical significance was not seen. The causative organisms remained similar in both groups as shown in Table 4. *Escherichia coli* was the prevalent pathogen in both groups.

Odds ratios with 95% CI were applied to both groups for clinical outcomes in patients with and without FN which also illustrated similar results as shown in Table 5. Although LBIs and MDIs were greater in number in febrile neutropenic patients in the no prophylaxis group, statistical significance was not found.

## Discussion

This study illustrated that levofloxacin prophylaxis resulted in a decrease in inpatient admissions due to suspected infections, and a decrease in FN episodes. These findings are in line with a study conducted in Saudi Arabia, which demonstrated a decrease in the duration of hospitalisation in patients receiving ciprofloxacin prophylaxis [14]. In contrast, a trial conducted by the Children’s Oncology Group showed that the mean duration of hospitalisation did not differ significantly between the two groups [16] which differs with the findings in our study. These results reflecting the effectiveness of antibacterial prophylaxis not only improves patient outcomes, but is also cost-effective, which is mainly of importance in LMIC settings where resources are scarce. A study was done on the cost-utility analysis by the SickKids team also revealed that antibacterial prophylaxis with levofloxacin in children receiving chemotherapy for AML or recurrent ALL is linked with significant cost reductions as compared to no prophylaxis [20].

The second important finding of our study was the significance of levofloxacin prophylaxis in the reduction of MDIs. Even though the population covered in this study was from LMIC was not relatable to that of the developed countries due to limited supportive care measures and the incidence of malnutrition is much higher, the results were similar [14–16, 21]. The study conducted at St. Jude Children’s Research Hospital also showed that patients who received any antibacterial prophylaxis were significantly less likely to have FN and infection-related adverse events than those who did not receive prophylaxis. Another study conducted from 2012 to 2015 at Dana Farber Cancer Institute (DFCI) also depicts similar results [16].

This study also highlighted the fact that the need for urgent interventions due to sepsis and septic shock was also significantly higher in those patients who were not on a prophylactic antibiotic. This would lead to fewer ICU admissions and further cost reduction. These facts were different from the study performed at St. Jude’s Hospital. The proportions of severe sepsis or sepsis requiring urgent intervention did not differ between the two groups as there was a different setting and lower incidence of infections as compared to developing countries [15]. A randomised controlled trial in Indonesia showed unexpected results. Patients in the ciprofloxacin arm were more likely to develop fever, clinical sepsis and death from complications than patients in the placebo arm. They discovered that the ciprofloxacin group had a lower absolute neutrophil count nadir. As a matter of fact, in developing countries malnutrition is also an important factor for fever and infections and prolonged neutropenia could add to the misery [22].

There was a trend toward a decreased rate of death during induction in the DFCI study, although this did not reach statistical significance [21] as our study also showed that overall mortality was similar in both groups. Apart from severe bacterial infections, numerous negative factors influenced the mortality results of acute leukaemia patients, including lethal haemorrhage, disseminated thrombosis and invasive fungal infections. As a result, we feel that the FN rate, bacteraemia rate and rate of MDIs were more important outcomes to assess the efficiency of levofloxacin prophylaxis rather than treatment-related mortality rates.

There were other studies which revealed strikingly different results from this study. A Canadian study showed although levofloxacin for FN prevention in patients with AML in adults was related to fewer hospitalisations but secondary outcomes such as total days of antibiotic treatment, day of readmission for FN and incidence of positive bacterial cultures were unaffected by levofloxacin [23]. The reason could be a small sample size and compliance to oral levofloxacin could not be assessed as it was retrospective study. Patel *et al* [24] also discovered that there was no significant difference in the FN, CDI, treatment antibiotic exposure days, or 30-day infection-related mortality between the two groups in AML and haematopoietic stem cell transplant patients [24]. This study was also retrospective single-centre study so results could not be compared effectively due to study design.

Studies regarding antibacterial resistance after levofloxacin prophylaxis have shown conflicting findings. The development of antibiotic resistance had reduced the use of antibacterial prophylaxis in a study performed in Australia [25] as fluoroquinolone resistance was higher to some extent in patients who were on levofloxacin prophylaxis but there was not any statistical difference in both groups similar to the results of our study. A meta-analysis discovered no statistically significant rise in quinolone-resistant infection, but the follow-up length in these trials was insufficient to address the issue of long-term resistance [13]. Few studies in adult cancer patients revealed similar facts. According to the Gruppo Italiano Malattie Ematologiche dell'Adulto (GIMEMA) Infection Program Data Center randomised placebo-controlled trial and a recent large meta-analysis of randomised controlled trials comparing fluoroquinolone prophylaxis with placebo or no intervention, fluoroquinolone resistance associated with prophylaxis seems not to effect clinical outcomes such as infection-related morbidity and mortality [26–29].

This study's nonrandomised design and comparison to historical controls are the key limitations in drawing concrete conclusions about the therapeutic efficacy of fluoroquinolone prophylaxis during induction chemotherapy for ALL. However, there was no significant demographic difference between both cohorts. The socioeconomic status, ALL induction chemotherapy protocol, supportive care services, intensive care management and empirical antibiotic treatment for FN as per the institutional guidelines were also similar in both groups. Infection control practices during both the time periods were evaluated. The incidence of central line blood stream infections, catheter associated urinary tract infections, hospital acquired infections, emergence of multi-drug resistant organisms and hand hygiene practices among doctors and allied medical staff remained same throughout both the study periods. Exposure to levofloxacin, on the other hand, relates to the risk of side effects such as *Clostridium difficile*-associated colitis, susceptibility to invasive fungal illness and musculoskeletal and neurological toxicity which were not collected in this study due to resource-limited setting.

## Conclusion

In conclusion, our study demonstrated that levofloxacin prophylaxis in HR paediatric ALL patients reduces the frequency and duration of inpatient hospital admissions and also reduces infection-related adverse events. Consequently, this is more cost-effective for the hospital and for the patient as well as there would be reduced time away from work. Levofloxacin can also play a role in the decline of infection-related adverse events and further critical interventions. This would be specifically beneficial in LMICs with limited supportive care services, high induction mortality and high rates of infections due to the logistic issues, lack of hygiene and literacy level of the population. Further prospective studies and randomised controlled trials are necessary for the use of levofloxacin prophylaxis regarding its beneficial effects and the long-term monitoring of the antibiotic susceptibility of the pathogenic organisms.

## Conflicts of interest and funding

There are no conflicts of interest and no financial support for this study.

## Figures and Tables

**Table 1.** Operational definitions (adapted from [15]).

Microbiologically documented infection	Diagnosed bacterial, viral, fungal or parasitic infection, with supportive microbiological evidence, such as a positive culture, antigen and antibody tests, clinical prodrome of some specific viral illnesses, laboratory and radiological evidence of fungal infection like beta-D glucan and galactomannan.
CDI	Infection diagnosed by the treating clinician for which a specific microbial cause could not be demonstrated.
LBI	Any infection with a microbiologically documented bacterial cause or that was clinically documented in categories typically attributed to bacterial infection, including pneumonia, skin and soft-tissue infection osteomyelitis or myositis, enterocolitis, otitis media or externa, sinusitis, epididymo-orchitis, central line infection, pharyngitis, perianal abscess or cellulitis, peritonitis, lymphadenitis or culture-negative sepsis [15].
BSI	Any infection caused by a recognised pathogen that was isolated from ≥1 blood culture in the context of a compatible clinical illness; common commensal bacteria were included if identified from multiple culture sets, or if a single blood culture set was collected before the start of antibiotic therapy and the result deemed clinically significant by the treating clinician.
Sepsis	The presence of two or more of the systemic inflammatory response syndrome criteria in patients with suspected infection.
Severe sepsis	Infection in conjunction with severe dysfunction of cardiovascular or respiratory systems or ≥2 other organ systems; fever or elevated WBC count not required.
Urgent intervention	Infection requiring admission to the intensive care unit (ICU), fluid resuscitation or supplemental oxygen or associated with clinician diagnosis of septic shock.
Drug resistance	Resistance to any drug as shown in the culture report of that patient.

**Table 2.** General characteristics of patients with or without prophylaxis.

Age group	Levofloxacin prophylaxis (*n* = 115)	No prophylaxis (*n* = 112)	*p*-value
≥10 years	58 (50.4%)	64 (57.1%)	0.311
<10 years	57 (49.6%)	48 (42.9%)	
Gender			
Male	75 (65.2%)	74 (66.1%)	0.892
Female	40 (34.8%)	38 (33.9%)	

Admitted in hospital			
Yes	55 (47.8%)	70 (62.5%)	**0.026**
No	60 (52.2%)	42 (37.5%)	

Body mass index Mean ± SD	14.6 ± 2.58	14.7 ± 2.48	0.647
Hospital stay (in days)			
≤3 days	17 (30.9%)	13 (18.6%)	0.109
4–7 days	18 (32.7%)	38 (54.3%)	**0.016**
>7 days	20 (36.4%)	19 (27.1%)	0.270
Re-admissions			
1	40 (72.7%)	52 (74.3%)	0.667
2	14 (25.5%)	15 (21.4%)	
3	1 (1.8%)	3 (4.3%)	
Risk profile			
T-ALL	30 (26%)	26 (23.2%)	0.364
B-ALL	85 (73.9%)	86 (76.8%)	0.364
t (9:22)	20 (23.5%)	23 (26.7%)	0.332
Hypodiploidy	15 (17.6%)	18 (20.9%)	0.323
MLL rearrangement	12 (14.1%)	10 (11.6%)	0.437
Prednisolone poor responder	35 (30.4%)	40 (35.7%)	0.241
CNS 3	32 (27.8%)	38 (33.9%)	0.197

Bold values denote a *p*-value < 0.005, which is considered significant.

**Table 3.** Clinical outcomes in patients with and without levofloxacin.

	Levofloxacin prophylaxis	No prophylaxis	*p*-value
Admitted in hospital	*n* = 55	*n* = 70	
Neutropenia			
FN	**31 (56.4%)**	**55 (78.6%)**	**0.008**
Non FN	24 (43.6%)	15 (21.4%)	
Infection-related adverse events			
CDI	22 (40.0%)	24 (34.3%)	0.510
MDI	9 (16.4%)	23 (32.9%)	**0.035**
BSI	1 (1.8%)	3 (4.3%)	0.810
LBI	30 (54.5%)	42 (60.0%)	0.540
Consequences of infection			
Sepsis	1 (1.8%)	6 (8.6%)	0.103
Death	4 (7.3%)	4 (5.7%)	0.454
Stable	50 (90.9%)	60 (85.7%)	0.375

Need for urgent interventions	14 (25.5%)	31 (44.3%)	**0.029**

Positive cultures	6 (10.9%)	19 (27.1%)	**0.030**
Organisms identified			
Gram-positive	1	5	
Gram-negative	3	14	
Both	2	0	
Drug-resistance			
Resistant to fluoroquinolone	5 (83.3%)	12 (63.1%)	0.355
Carbapenem-resistant enteric rods	2 (33.3%)	8 (42.1%)	0.544
Vancomycin-resistant organism	1 (16.6%)	2 (10.5%)	0.578
Multidrug resistant organism	2 (33.3%)	2 (10.5%)	0.233

Bold values denote a *p*-value < 0.005, which is considered significant.

**Table 4.** Pattern and sites of isolated organisms.

Isolated organisms	Levofloxacin prophylaxis (*n* = 9)	No prophylaxis (*n* = 23)
*Bacteria*		
* E. coli*	2	6
* K. pneumoniae*	1	4
* Acinetobacter baumannii*	1	1
* Pseudomonas aeruginosa*		2
* Burkholderia*		1
* S. aureus*	1	1
Coagulase-negative Staphylococci		2
* Enterococcus*	1	2
* M. tuberculosis*		1
Fungi		
* Aspergillus*		1
* Candida*	1	
Viruses		
Varicella	1	1
Measles		1
Herpes	1	
Site of isolate		
Blood culture	3	12
Urine culture	1	4
Pus culture	2	3
Central line		1

Bold values denote a *p*-value < 0.005, which is considered significant.

**Table 5.** Risk factor (odds ratio) with and without prophylaxis.

	Levofloxacin prophylaxis (*n* = 55)	No prophylaxis (*n* = 70)	OR (95% of CI)	*p*-value
Outcome				
FN	**31 (56.4%)**	**55 (78.6%)**	**0.35 (0.15–0.82)**	**0.008**
FN with CDI	10 (32.3%)	16 (29.1%)	1.16 (0.40–3.33)	0.758
FN with MDI	9 (29.0%)	21 (38.2%)	0.66 (0.23–1.88)	0.393
FN with LBI	18 (58.1%)	35 (63.6%)	0.79 (0.29–2.14)	0.609

CDI	22 (40.0%)	24 (34.3%)	1.28 (0.58–2.83)	0.510
MDI	**9 (16.4%)**	**23 (32.9%)**	**0.40 (0.15–1.03)**	**0.035**
LBI	30 (54.5%)	42 (60.0%)	0.80 (0.37–1.74)	0.540

Bold values denote a *p*-value < 0.005, which is considered significant.
